# Rural-urban classification is associated with patient- and population-level disparities in leukemia relative to other pediatric cancers

**DOI:** 10.3934/publichealth.2026025

**Published:** 2026-04-15

**Authors:** Benjamin N. Vickers, April R. Jimenez, Kristin S. Bogda, Elizabeth Y. Jimenez, Tracie C. Collins

**Affiliations:** 1 College of Population Health, The University of New Mexico, Albuquerque, NM, United States; 2 Exercise and Sport Sciences Department, New Mexico Highlands University, Las Vegas, NM, United States; 3 Department of Health, Human Performance and Recreation, College of Education and Health Professions, University of Arkansas, Fayetteville, AR, United States

**Keywords:** pediatric leukemia, pediatric cancer, community exposure, rural, urban, metropolitan areas, multilevel analysis, natural counterfactual mediation, associational mediation, case scores

## Abstract

**Background:**

Despite leukemia being the most commonly diagnosed form of pediatric cancer, prior surveillance research has not linked the odds of a given pediatric cancer being leukemia to rural–urban county designations.

**Purpose:**

We sought to elucidate the patient-level role of rural–urban county designations in predicting the odds of a given pediatric cancer diagnosis being leukemia while observing the role of these county designations in conveying leukemia/non-leukemia disparities in the U.S. pediatric cancer population.

**Methods:**

The sample included pediatric cancer diagnoses from the Surveillance, Epidemiology, and End Results (SEER) 21 dataset from 2010 through 2017. The study outcome was binary; cases were leukemia and controls were non-leukemia. The focal predictor was the rural–urban county designation. The analysis used patient- and community-level covariates in a logistic regression model to identify the role of rural–urban designations in predicting a leukemia diagnosis. The model also generated case scores, which were the population probabilities of diagnoses being leukemia, and used these to conduct mediation analyses to determine the population-average associations of the rural–urban designations with leukemia.

**Results:**

In the adjusted model, rural counties adjacent to metropolitan areas (henceforth, “metros”) had a significant association with leukemia cases, with 29.4% greater odds compared to the largest metro counties. In the population-level mediation via case scores, not only rural counties adjacent to metros, but also medium and small metros, had greater population-average odds of leukemia compared to the largest metros.

**Conclusions:**

While only rural counties adjacent to metros were consequential for modeling leukemia diagnoses among patients, population-average disparities in leukemia odds were also apparent in small and medium metros compared to the largest metros. This may indicate that leukemia risk exposures, existing systematically in rural counties adjacent to metros, may also be present, to lesser extents, in the small and medium metros.

## 1. Introduction

Leukemia is the leading type of malignant pediatric cancer in the zero-to-14-year age range [1]. Acute lymphocytic leukemia (ALL) accounts for an estimated 26% of all new malignant pediatric cases in the United States, and acute myeloid leukemia (AML) accounts for an additional 5% [1]. Although not as comparatively consequential in the 15-to-19-year age range, ALL and AML together account for an estimated 12% of new malignant cancers for adolescents in the United States [1].

Pediatric leukemias in the U.S. have been linked to genetics, health behaviors, and environmental exposures [2]–[4]. Environmental exposures associated with leukemia risk, such as solvents, pesticides, other chemicals, ionizing radiation, and traffic-caused air pollution, can differ by rurality (i.e., rural or urban residence) [5],[6]. For example, individuals living in rural areas focused on agricultural production have greater exposure to pesticides [7], while individuals living in more urban areas may have more exposure to traffic-related air pollution [8], resulting in disparities in associated health outcomes. Little U.S. research has examined relationships between pediatric leukemia risk and rural–urban residence (i.e., rurality). Adelman et al. [9] examined population-based leukemia incidence rates in U.S. children aged zero to four years related to rurality using U.S. Department of Agriculture RUC Codes. They found that incidence rates were significantly lower in rural areas not adjacent to metropolitan areas (henceforth, “metros”), but all areas in or adjacent to metros had statistically indistinguishable incidence rates. This study concluded that one or more risk factors for pediatric leukemia in young children were largely absent in areas geographically isolated from U.S. metropolitan areas [9]. Even so, U.S. surveillance data have shown a slight transition of leukemia-associated risk in the first two decades of the 21^st^ century away from urban areas and toward rural areas [10]. However, the overall results have been mixed, and this is further complicated by natural variations in geography, in cultural-environmental exposures, and in measurement methods used with different national populations [11].

Therefore, the aim of this study was to simultaneously elucidate the patient-level and population-level relevance of the rural–urban context for identifying the kinds of communities where pediatric cancers are more likely to be leukemia than other kinds of pediatric cancers. Based on the Adelman study [9], we hypothesized that patient residence in rural counties in the U.S. adjacent to urban areas would have a stronger multilevel-adjusted patient-level association with the likelihood of a pediatric cancer being diagnosed as leukemia and/or a stronger population-average association with the same outcome mediated by disparities in the modeled patient-level probabilities between rural–urban designations.

## 2. Materials and methods

### 2.1. Study design

This study employed a secondary analysis of cancer registry data. Since the outcome variable and exposure/independent variables were all assessed and valid at the time of each cancer diagnosis, the study design was cross-sectional with a cohort framing to account for data collected over several years. However, since the outcome variable was binary, we used case-control terminology and odds ratios (OR) to assess the associations between independent variables and the outcome categories.

### 2.2. Data source

The data used for this secondary analysis were from the National Cancer Institute's (NCI) Surveillance, Epidemiology, and End Results (SEER) cancer registry. The SEER 21 dataset included cancer registries from 21 different reporting sources (see Note†). The SEER 21 registry encompassed all cancer diagnoses within the geographic coverage areas for each of the 21 reporting sources.

### 2.3. Study sample

The November 2019 version of the SEER 21 cancer registry dataset included 9,821,960 records across 18 years (2000–2017). The study sample was a subset of the SEER 21 dataset. Initially, we limited the sample to patients who were diagnosed when younger than 20 years of age. Restricting the dataset to this age range, the data were reduced to 97,998 records, which was 1% (0.998%) of the records in the full dataset. This is consistent with data from the National Cancer Institute showing that approximately 1% of all cancer diagnoses occur in patients under 20 years of age [12]. We then limited the dataset to diagnosis years from 2010 through 2017, as there were significant changes to leukemia diagnostic coding starting January 1, 2010 [13]. In the resulting sample, the greatest percentage of missing values among the study variables was for the rural–urban category (0.21% missing). Given the negligible percentage of missing data, records with missing values were excluded from the analysis, resulting in a final analytic sample of 44,716 pediatric cancer diagnoses. This included 4328 cases (9.68%) with a leukemia diagnostic code, and 40,388 controls with other cancer diagnoses. In the post hoc power analysis for a multivariable generalized linear model, a power of 0.8 and a Type 1 error probability (α) of 0.05 would require a sample size of 2170 to detect a multiple correlation coefficient of 0.1 [14],[15] while accounting for 22 possible model parameters tied to the eight model independent variables that were under consideration.

### 2.4. Variables

The study outcome was a binary variable indicating whether the pediatric patient with cancer was diagnosed with leukemia or not. The variable was considered a *case* for a leukemia diagnosis (ICD-O-3 histology codes 9800–9949) and a *control* for any other non-leukemia cancer diagnosis. The rural–urban variable was geographically linked to each cancer diagnosis as an ecological, community-based factor. It included five nominal categories based on the U.S. Department of Agriculture's [16] Rural–Urban Continuum Codes (RUCCs): nonmetro county not adjacent to metro county, nonmetro county adjacent to metro county, county of a metro with population <250,000, county of a metro with population 250,000–1,000,000, and county of a metro with population >1 million. A nonmetro county is considered “adjacent” if it shares at least one common boundary with a metro county and 2% or greater of that nonmetro county's workforce commutes to central metro counties [16].

Seven additional variables, at both the patient level and community level, were included as covariates. This multilevel approach to covariate adjustment alongside rural–urban designations as the focal independent variable within a single cancer study intentionally followed the prescribed methodological recommendation of Meilleur et al. [17]. Community-level variables were inflation-adjusted (2018 U.S. Dollar) median household income categories of the residential county (<$45,000, $45,000 to $50,000, $50,000 to $55,000, $55,000 to $60,000, $60,000 to $65,000, $65,000 to $70,000, $70,000 to $75,000, >$75,000+) and a binary purchased/referred care delivery area (PRCDA) variable (“1” for any county containing all or part of a tribal land/reservation or sharing a common boundary with the same, “0” for all other counties). Patient sociodemographic variables included age in years, binary sex, and ethnicity/race in four categories: Hispanic of any race, non-Hispanic Black, non-Hispanic White, and non-Hispanic of other or unknown race. While age and sex might have both biological and social implications for pediatric cancer diagnoses, the ethnicity/race variable was included to account only for health outcome disparities in groups that have been economically and socially marginalized and not as a biological variable. Other patient-level variables included the calendar year of diagnosis (range: 2010–2017) and the diagnosis reporting source (“1” for hospital inpatient/outpatient or physician's office, “0” for other sources, such as a cancer care center).

### 2.5. Data analysis

Descriptive statistics were calculated for all variables by case-control status. To assess the bivariate association between each variable and the outcome, we performed the chi-square test for nominal independent variables and the Cochran–Armitage test for the single ordinal independent variable: median county household income.

Logistic regression was used to identify the best-fitting multilevel model of the odds of a leukemia case using the eight variables. The operational coding and levels of measurement of the variables were chosen based on their contributions to improved model fit using the Akaike Information Criterion (AIC), and all eight variables were needed to achieve the best fit. Given that the community-level rural-urban variable could conceivably better function as a clustering variable to condition patient-level covariates rather than functioning as an adjusted variable alongside covariates, we also generated mixed effects models with which to compare the predictive accuracy of the fixed effects best-fitting logistic model. Compared to models with random intercepts and slopes selected using fit statistics with rural-urban clusters, the fixed effect logistic model was the most predictive according to mean absolute errors. While using community-level predictors alongside patient-level predictors within a multilevel fixed effects model could introduce ecological errors that might be mitigated by random effects in a mixed model, the sole purpose of the community-level predictors in the patient-level model was to enhance patient-level prediction. In this way, the community-level fixed effects would be only the average community-level effect for patients after adjusting for the other predictors. A sensitivity analysis was conducted with the lymphoid and myeloid leukemia subtypes.

Last, in order to accurately assess a possible population-level impact of rural–urban designations in differentiating leukemia cases from non-leukemia controls among U.S. pediatric cancer diagnoses, the set of probabilities generated from the logistic model was used as leukemia case scores (i.e., percentage probabilities that a diagnosis would be a case). In the population-based sample, the crude odds ratio associations of the four rural–urban designations with case-control status, contrasted with the reference large metro category, represented the overall expected associations of these rural–urban designations with case-control status when averaged among their respective patient populations. However, due to the aggregation of all pediatric cancer diagnoses at the rural–urban designation level, these population-average associations can be distorted when a rural–urban category might be either similarly or oppositely related to risk exposures for both cases and controls.

To account for these possibilities, we disaggregated the crude population-level associations into a naturally occurring case score component and a residual component. This disaggregation, or decomposition into two parts, was accomplished using natural counterfactual mediation, which utilizes generalized linear models and formulas developed by VanderWeele and colleagues [18]–[20]. While these mediation techniques were developed with causal analysis in mind, they can similarly be used to assess “associational mediation”, which is an observation of the extent to which an association between two variables can be statistically accounted for, naturally in the population, by a third variable. This natural counterfactual mediation technique sets the mean value of the case scores for each of the rural–urban designations as the naturally occurring value of the case score mediator for the population of each rural–urban designation. Disaggregating the crude population-level associations between rural–urban designations and case-control statuses into case score components and residual “direct” components allows observation of the true population-level roles of the rural–urban designations in differentiating between cases and controls, undistorted by population aggregation.

All statistical analyses were performed using SAS software, version 9.4 (SAS Institute Inc., Cary, NC). Any p-value less than 0.05 was considered statistically significant for the descriptive bivariate tests. For the logistic regression model, there were 22 parameters across the eight variables. The five categories of the rural–urban variable required four model parameter tests; the reference group included counties encompassing metropolitan areas with over 1 million residents. Therefore, a Bonferroni adjustment to the Type 1 error probability was used to compare these four parameter tests (α_*B*_ = 0.05/4 = 0.0125) to the single, global chi-square test of bivariate association between the rural–urban variable and case-control status [21]. Although the covariates were included in the model only to improve model fit and predictive accuracy, Bonferroni adjustments were likewise made for multiple comparisons among covariate groups for consistency.

## 3. Results

Table 1 shows the counts and percentages of cancer diagnoses for the rural–urban county designations and the covariates by case-control status. All eight independent variables were associated with case-control status. The global bivariate association of rural–urban designations with case-control status was significant (*p* = 0.023). This was primarily driven by differences between non-metro counties adjacent to metros (proportion of cancer diagnoses being leukemia cases: 6.15%, controls: 5.01%) and metros with a population greater than 1 million (cases: 63.98%, controls: 65.27%).

Table 2 includes the results of the multilevel logistic regression model. Only children residing in a non-metro county adjacent to a metro had significantly higher mean odds of being diagnosed with leukemia compared to children living in the largest metropolitan areas (OR 1.294, *p* < 0.001), after adjusting for other variables in the model. No other rural–urban designations significantly distinguished between case and control status in the model. The other community-level variables were not associated with case-control status.

In the sensitivity analysis by subtype, 66.8% of the cases were lymphoid leukemia, 29.9% were myeloid leukemia, and the remaining roughly 3% were “other” or “not otherwise specified” and were excluded from the sensitivity analysis. The multilevel model result (Table 2) for the rural–urban categories was robust to the lymphoid and myeloid subtypes. However, the odds ratio for nonmetro adjacent to metro vis-a-vis the reference large metro was slightly larger (38% greater odds) among the lymphoid leukemias compared to the myeloid leukemias (22% greater odds), both in comparison to all non-leukemia controls.

**Table 1.** Descriptive statistics: pediatric (<0 years) leukemia diagnoses (cases) and non-leukemia cancer diagnoses (controls) by rural–urban county designation and the covariates (SEER 21, 2010–2017, n = 44,716 diagnoses with no missing data).

Independent variables	Leukemia cases n = 4328 (9.68%)	Non-leukemia controls n = 40,388 (90.32%)	Test statistic^1^ and *p*-value

	count (column %)	count (column %)	
Rural–urban county designation^2^			{χ^2^ = 11.38} 0.023
Nonmetro: not next to metro	144 (3.33%)	1339 (3.32%)	
Nonmetro: next to metro	266 (6.15%)	2025 (5.01%)	
Metro: <250,000	291 (6.72%)	2594 (6.42%)	
Metro: 250,000–1 million	858 (19.82%)	8067 (19.97%)	
Metro: >1 million	2769 (63.98%)	26,363 (65.27%)	
Age category^3^			{χ^2^ = 81.15} <0.001
<1 year	310 (7.16%)	2473 (6.12%)	
1–4 years	968 (22.37%)	9353 (23.16%)	
5–9 years	820 (18.95%)	6884 (17.04%)	
10–14 years	1016 (23.48%)	8010 (19.83%)	
15–19 years	1214 (28.05%)	13,668 (33.84%)	
Sex^3^			{χ^2^ = 75.54} <0.001
Male	2558 (59.10%)	21,068 (52.16%)	
Female	1770 (40.90%)	19,320 (47.84%)	
Ethnicity/race^3^			{χ^2^ = 65.97} <0.001
Hispanic (any race)	1255 (29.00%)	11,205 (27.74%)	
Non-Hispanic (NH) black	567 (13.10%)	4189 (10.37%)	
Non-Hispanic (NH) white	2024 (46.77%)	21,188 (52.46%)	
NH (other/unknown race)	482 (11.14%)	3806 (9.42%)	
County contains or is next to tribal land [PRCDA]^4^			{χ^2^ = 4.39} 0.036
Yes	1270 (29.34%)	12,476 (30.89%)	
No	3058 (70.66%)	27,912 (69.11%)	
County median household income (inflation-adjusted)^4^			{Z = 3.47} <0.001
<$45,000	431 (9.96%)	3678 (9.11%)	
$45,000–$49,999	304 (7.02%)	2552 (6.32%)	
$50,000–$54,999	436 (10.07%)	4039 (10.00%)	
$55,000–$59,999	535 (12.36%)	4985 (12.34%)	
$60,000–$64,999	812 (18.76%)	7167 (17.75%)	
$65,000–$69,999	376 (8.69%)	3736 (9.25%)	
$70,000–$74,999	291 (6.72%)	2555 (6.33%)	
≥$75,000	1143 (26.41%)	11,676 (28.91%)	
Reporting source^3^			{χ^2^ = 24.46} <0.001
Hospital or clinic	4184 (96.67%)	38,357 (94.97%)	
Other (e.g., cancer center)	144 (3.33%)	2031 (5.03%)	
Year of diagnosis^3^			{χ^2^ = 16.64} 0.020
2010	536 (12.38%)	4944 (12.24%)	
2011	500 (11.55%)	5070 (12.55%)	
2012	519 (11.99%)	5043 (12.49%)	
2013	566 (13.08%)	4962 (12.29%)	
2014	564 (13.03%)	5097 (12.62%)	
2015	518 (11.97%)	5298 (13.12%)	
2016	543 (12.55%)	5103 (12.63%)	
2017	582 (13.45%)	4871 (12.06%)	

Note: ^1^ The Cochran–Armitage test was used for the ordinal variable median county household income, and the chi-square test was used for all other nominal variables. The global associational test for nominal variables (chi-square) had a null hypothesis of no differences in any of the proportions of cases and controls among the nominal categories versus an alternative hypothesis of any difference in proportions. The global associational test for the ordinal variable (Cochran–Armitage test) had a null hypothesis in which the proportions of cases and controls are the same across all ordered levels of median county household income. The alternative hypothesis is that there is a linear trend (either increasing or decreasing) in the proportions of cases and controls across the ordered categories. ^2^ 0.21% of observations missing. ^3^ No missing data. ^4^ 0.02% of observations missing.

**Table 2.** Multilevel (patient- and community-level predictors) logistic regression model: model parameter estimates with standard error (S.E.) and adjusted odds ratios for rural–urban county designation and the covariate associations with pediatric (<20 years) leukemia cases versus non-leukemia cancer controls (SEER 21, 2010–2017, n = 44,716).

Independent variables	Model estimate (S.E.)	Adjusted odds ratio	*p*-value
Intercept	−2.694 (0.123)		<0.001
Rural–urban county status (reference: metro >1 million) (α_B_ = 0.05/4 = 0.0125)			
Nonmetro: not next to metro	0.027 (0.096)	1.027	0.779
Nonmetro: next to metro	0.258 (0.075)	1.294	<0.001^1^
Metro: <250,000	0.072 (0.070)	1.075	0.299
Metro: 250,000–1 million	0.035 (0.042)	1.036	0.408
Patient-level covariates
Age category (reference: <1 year) (α_B_ = 0.05/4 = 0.0125)			
1–4 years	−0.199 (0.069)	0.819	0.004^1^
5–9 years	−0.058 (0.071)	0.943	0.411
10–14 years	0.013 (0.069)	1.013	0.850
15–19 years	−0.324 (0.068)	0.723	<0.001^1^
Sex (none missing) (reference: female) (α_B_ = 0.05/1 = 0.05)		
Male	0.278 (0.033)	1.320	<0.001^1^
Ethnicity/race (reference: Non-Hispanic White) (α_B_ = 0.05/3 = 0.0167)		
Hispanic (any race)	0.159 (0.039)	1.172	<0.001^1^
Non-Hispanic (NH) black	0.333 (0.052)	1.395	<0.001^1^
NH (other/unknown race)	0.313 (0.055)	1.368	<0.001^1^
Community-level covariates
Reporting source (reference: other, e.g., cancer center) (α_B_ = 0.05/1 = 0.05)			
Hospital or clinic	0.418 (0.088)	1.518	<0.001^1^
Year of diagnosis (reference: 2010) (α_B_ = 0.05/7 = 0.0071)			
2011	−0.085 (0.066)	0.919	0.195
2012	−0.052 (0.065)	0.950	0.428
2013	0.044 (0.064)	1.044	0.496
2014	0.015 (0.064)	1.015	0.812
2015	−0.099 (0.065)	0.906	0.127
2016	−0.018 (0.064)	0.982	0.783
2017	0.098 (0.063)	1.103	0.122

Note: ^1^ Significant *p*-value < α_*B*_ = with Bonferroni adjustment for multiple tests.

The natural levels of the case score mediator for the populations of each rural–urban designation were the mean case scores shown in Table 3. The mean case score for the sample was 9.68%, which exactly matched the leukemia proportion in the sample. Case scores ranged from 3.65% to 21.47% with a standard deviation of 2.35 percentage points. Three of the four rural–urban designations had population-average associations with case-control status that were significantly mediated by their respective natural case scores when contrasted with the largest metros. Most notably, the crude population-average case odds for nonmetro counties adjacent to metros were significantly greater by 23.7% compared to the crude population-average case odds for the largest metros. However, when mediating this crude association by case scores, the population-average case odds were 29.8% greater for nonmetro counties adjacent to metros compared to the largest metros. The lower crude association of 23.7% greater odds was due to the residual direct effects that tended non-significantly toward controls. So, while the odds of a leukemia diagnosis were certainly greater among the population of pediatric cancer diagnoses in rural areas adjacent to urban areas compared to those of large urban areas via the case scores, there was not a systematic residual association between these two populations and case-control status after accounting for case scores.

**Table 3.** Leukemia case score associational mediation: rural–urban designation of patient county of residence at time of pediatric (<20 years) cancer diagnosis compared with counties of metros with more than one million residents for their comparative associations with leukemia cases versus non-leukemia controls using odds ratios (OR). Total associations decomposed into case score-mediated and residual direct associations (SEER 21 data, 2010–2017, n = 44,716).

(p-values in parentheses)	OR _Total association_	OR _Natural mediated_	Mean case score (%)	OR _Natural direct_
Nonmetro not next to metro	1.019 (0.837)	1.004 (0.600)	9.711	1.014 (0.875)
Nonmetro next to metro	1.237 (0.006)^1^	1.298 (<0.001)^1^	11.611	0.953 (0.563)
Metro: <250,000	1.068 (0.329)	1.083 (<0.001)^1^	10.087	0.987 (0.842)
Metro: 250,000–1 million	1.014 (0.744)	1.012 (<0.001)^1^	9.615	1.002 (0.968)
Reference: metro: >1 million			9.506	

Note: ^1^Significant *p*-value < (α_*B*_ = 0.05/4 = 0.0125) with Bonferroni adjustment for multiple tests.

Additionally, although the other three rural–urban designations were not significantly associated with case-control status in the adjusted model, two of them were significantly associated with the odds of a leukemia diagnosis at the population-average level, when accounting for natural case scores. The population-average case score for metros with less than 250,000 residents had 8.3% greater leukemia odds than did the population-average case score for large metros. Additionally, although small in effect size, the population-average case score for metros with a population between 250,000 and 1 million residents had 1.2% greater leukemia odds compared to the largest metros. The population-average case score for nonmetro counties not adjacent to metros had case odds that were not significantly different from those of the largest metros.

Taking these results together, we observed an increasing dose-response relationship in population-average leukemia odds from large metros, at minimum, toward nonmetro counties adjacent to metros, at maximum. As with rural counties adjacent to metropolitan areas, the population-average tendencies of small and medium metropolitan areas toward leukemia cases did not exhibit significant residual associations with case-control status. This indicated that these population-average associations with case-control status, which were apparent in small and medium metros when compared to large metros, were only systematically apparent through, or mediated by, modeled case scores.

## 4. Discussion

This study employed a novel approach in risk factor epidemiology using a large, population-comprehensive cancer registry. Due to each unit of analysis being a single reported pediatric cancer diagnosis, the purpose of the study was to assess the extent to which the diagnosis-linked rural–urban designation of the patient's county of residence in the United States was systematically associated with the probability that the child would be a leukemia case rather than a non-leukemia control. Based on predictive accuracy, the rural–urban designation of the county of residence performed best as an adjusted factor in a patient-level logistic regression model than as a clustering factor for random effects in mixed models. Using this logistic regression model, residence in nonmetro counties adjacent to metros was associated on average with 29.4% greater odds of leukemia compared to the largest metros (population >1 million). After generating leukemia case scores (i.e., probabilities that a diagnosis would be a leukemia case) for the sample from the logistic regression model, three of the rural–urban county designations had a significant population-average association with case-control status when mediated by natural case scores. While there were no significant differences in odds between the populations of the largest metros and nonmetro counties that were not adjacent to metros, there was a significant dose-response increase in case score-mediated odds from large metros to medium metros to small metros to nonmetro counties adjacent to metros. After accounting for these case score-mediated associations, there were no significant residual associations between the rural–urban designations and case-control status at the population level.

Taking all these results together, we observed that the community-level factor of rural–urban designation, linked to pediatric cancer diagnoses by residential county in the U.S., contributed to both patient-level odds and collective population-level disparities in the type of cancer diagnosis received. The main patient-level implication was that rather than serving as a cluster in which other risk factors are moderated, the community-level designation of rural areas adjacent to urban areas functioned for patients as a unique risk factor for a pediatric cancer being leukemia, independent of other noteworthy patient-level risk factors like age, ethnicity/race, and sex. Even so, this was a mean effect and not to be attributed homogenously to residents within these communities. Given our knowledge of the role of environmental exposures for leukemia risk from prior research, it is logical to hypothesize that rural areas adjacent to urban areas systematically represent, to some extent, an environmental exposure profile that more closely aligns with leukemia risk than with the risk profiles of other pediatric cancers. This does not mean that risk exposures for other cancers are not also present in rural areas adjacent to urban areas or that leukemia risk exposures are not also present in other rural–urban designations, but it rather indicates that the relative contrast between these exposures in rural areas adjacent to urban areas is more consistently differentiated than in other kinds of rural–urban designations.

The main population-level implication was that all rural–urban designations, except those rural areas not adjacent to urban areas, had greater population-average leukemia odds due to unequal natural leukemia case scores within these populations compared to the largest metros. Furthermore, apart from the multivariable-determined leukemia case scores, there were no other “residual” ways apparent in which rural–urban designations were significantly associated with the odds that typical pediatric cancer diagnoses across these area-based populations would be leukemia. Therefore, not only rural counties adjacent to metros, but to a lesser extent also small and medium metros, more consistently encapsulate the total risk profile that tends toward a pediatric cancer being leukemia. Yet, at the patient level, there was something meaningful about the exposure of residing in rural counties adjacent to metros, specifically, that contributed to greater leukemia odds when combined with the contributions from other significant patient-level independent variables.

In prior research, evidence of differentiation of both cancer incidence and mortality risk by cancer type and rural–urban designation was observed. For example, Blake et al. [22] found higher incidence and mortality rates among “rural” (i.e., nonmetro, based on RUCCs) populations of adults for certain types of cancers, such as “...cervical cancer (measured among women only) as well as colorectal, kidney, lung and bronchus, melanoma, and oropharyngeal cancers”. On the other hand, they found higher incidence rates among metro adult populations for liver, thyroid, and breast cancers [22]. Similarly, among nonmetro areas with urbanized (i.e., densely populated) populations of 2500 or more, they observed higher cancer incidence rates for those counties that were adjacent to metro areas [22]. Similar research among pediatric cancer populations is lacking. Even so, taking those findings together with the present study, the adjacency of nonmetro communities to metros appears to pose contextual and/or environmental risks for some cancers, including pediatric leukemia. Additionally, while the sensitivity analysis showed the nonmetro adjacent to metro result to be robust to both lymphoid and myeloid leukemia subtypes, the fact that the average association was slightly stronger for lymphoid leukemia was noteworthy. The lymphoid subtype accounts for a strong majority of the pediatric cases, and given its crucial role in the immune system (i.e., lymphocytes), the noted result points to a hypothesized role of environmental exposures systematically located in nonmetro areas next to metro areas driving specifically immune responses in children, perhaps in complex interaction with genetic factors, that lead to leukemogenesis [11].

While the results were consistent and robust to leukemia subtype, they were also modest and point toward a need for further studies that can identify the stronger and more proximal drivers of the macro association of the rural–urban context with a differentiation among the types of pediatric cancers being diagnosed. Future research should explore the possible links between the established environmental risk factors for pediatric leukemia and these non-metro communities adjacent to metros. While the present study included some of the current knowledge on environmental risk factors for pediatric leukemia in the introduction, commenting further on how those risk factors might be linked to the rural–urban findings in this study is beyond the scope of this study. Environmental risk factors were not available in the SEER dataset.

The external validity of this study for the U.S. pediatric cancer patient population was strong, and there is corroborating evidence that the RUCCs urban/rural population percentages that occur in SEER registry reporting jurisdictions were acceptable representations of these categories in the U.S. population [22]. Although the data were somewhat dated, the year of diagnosis was not associated with the outcome from 2010 to 2017, indicating that the results were robust across the study timeframe and likely relevant beyond 2017. Furthermore, these data were valid for demonstrating our use of natural, counterfactual mediation techniques to present a population-average association of a community-level factor with a patient-level outcome undistorted by artifactual noise in the data that is irrelevant to the multilevel-determined patient model, which is a novel advancement for future multilevel, population-based epidemiological studies.

The main limitation of this study was that it had a cross-sectional design among pediatric cancer diagnoses, and therefore, it did not address incidence rates or population risk. Nonetheless, this study has briefly discussed peer-reviewed research on pediatric leukemia risk factors in an effort to provide a broader context for its findings. Even so, we were unable to directly link the established environmental risk factors to rural–urban categories, and this could introduce a risk of uncontrolled confounding by these factors if they vary by geography. Future research will be needed to determine if higher levels of certain environmental pediatric leukemia risk factors are indeed systematically present in rural areas adjacent to urban areas. Another limitation is that while the rural–urban designations were linked to diagnoses by patient county of residence, we did not have access to the specific counties in order to protect patient privacy, so we could not account for within-county autocorrelations. To address this, future research should not only identify environmental pediatric leukemia risk factors common to rural communities adjacent to urban areas but also attempt to measure meaningful variations of these common exposures between these communities in an attempt to further refine and calibrate their environmental risk profiles.

Future research should also examine the role of rural–urban residential designations in adult leukemia diagnoses. While leukemia is the most common type of pediatric cancer, the risk of leukemia incidence actually increases with age, and older adults between 65 and 74 years of age are the most at risk of being diagnosed with leukemia [23]. In both pediatric and adult populations, longitudinal area-based leukemia risk analyses that account for rural–urban designations should also be conducted; this would require special access to more restrictive SEER databases rather than the standard public use files. Additionally, other conceptualizations and operationalizations for the concept of rurality, for example, should be explored for their potential to better capture systematic community-based exposures and risks for leukemia [24]. Last, and most importantly, environmental science should explore the systematic existence, quantities, and distributions of both documented and new environmental risk factors for leukemia that may especially occur in rural areas adjacent to urban areas, but may also be found in the more rural parts of smaller urban areas.

## 5. Conclusions

These findings, coupled with further research to link risk factors to residence in rural areas adjacent to urban areas, can not only help further refine the environmental risk factor profile for pediatric leukemia but also provide a crucial clue as to the *kinds* of communities where these risk profiles are heightened. Additionally, this enhancement to the risk factor profile will assist with identifying public health interventions not only for rural areas adjacent to urban areas but also for other kinds of communities where these risk factors might occur. In addition to public health efforts, these findings might also provide clues to enhance clinical cancer research, screenings, and other clinical interventions, as well as other preventive efforts in areas like public policy. Public health investigations should explore and identify the environmental and social-contextual exposures among pediatric leukemia patients that are unique to their lives in nonmetro counties adjacent to metros. Additionally, the innovative multilevel methods used in this study can be used with other cancers and patient ages in the U.S. population and beyond.

## Use of AI tools declaration

The authors declare they have not used Artificial Intelligence (AI) tools in the creation of this article.
